# Lung anatomy and histology of the extant coelacanth shed light on the loss of air-breathing during deep-water adaptation in actinistians

**DOI:** 10.1098/rsos.161030

**Published:** 2017-03-08

**Authors:** Camila Cupello, François J. Meunier, Marc Herbin, Gaël Clément, Paulo M. Brito

**Affiliations:** 1 Departamento de Zoologia, Universidade do Estado do Rio de Janeiro, R. São Francisco Xavier, 524-Maracanã, Rio de Janeiro 20550-900, Brazil; 2 Département des Milieux et Peuplements Aquatiques, UMR BOREA 7208, Sorbonne Universités-MNHN-UPMC-CNRS-IRD, Muséum national d'Histoire naturelle, 57 rue Cuvier, Paris 75231, France; 3 Département Écologie et Gestion de la Biodiversité, UMR MECADEV 7179, Sorbonne Universités-MNHN-CNRS, Muséum national d'Histoire naturelle, 57 rue Cuvier, Paris 75231, France; 4 Département Histoire de la Terre, UMR CR2P 7207, Sorbonne Universités-MNHN-UPMC-CNRS, Muséum national d'Histoire naturelle, 57 rue Cuvier, CP38, Paris 75005, France

**Keywords:** Actinistia, *Latimeria chalumnae*, air-breathing organ, lung, histology, ontogeny

## Abstract

Lungs are specialized organs originated from the posterior pharyngeal cavity and considered as plesiomorphic for osteichthyans, as they are found in extant basal actinopterygians (i.e. *Polypterus*) and in all major groups of extant sarcopterygians. The presence of a vestigial lung in adult stages of the extant coelacanth *Latimeria chalumnae* is the result of allometric growth during ontogeny, in relation with long-time adaptation to deep water. Here, we present the first detailed histological and anatomical description of the lung of *Latimeria chalumnae*, providing new insights into its arrested differentiation in an air-breathing complex, mainly represented by the absence of pneumocytes and of compartmentalization in the latest ontogenetic stages.

## 1. Introduction

The specialization for air-breathing may have arisen in fishes during the Silurian (from 438 million to 408 million years ago) [1–3], some millions of years before the origin and terrestrialization of the first tetrapods [4–11]. This specialization has probably emerged independently, as many structures (such as lung, skin and gills) named as air-breathing organs (ABOs) [1] can be involved in this process.

Osteichthyans present specialized organs that are originated from the posterior pharyngeal cavity [3,12], such as lungs and gas bladders, to which the homology is still debatable. Lungs, as a ventral derivate, are considered as plesiomorphic for osteichthyans [11,13], as they are found in the extant basal actinopterygian polypterids [14–16], and in all major groups of extant sarcopterygians: coelacanths [17,18], lungfishes [1,19–21] and tetrapods.

A pulmonary apparatus was described for fossil coelacanths [17], and the presence of a vestigial lung in the extant coelacanth *Latimeria chalumnae* Smith, 1939 [22,23], has been recently confirmed [18]. However, the histological anatomy of the extant coelacanth lung still needs to be fully described, as few studies about the anatomy and histology of this organ have been made in this lobe-finned sarcopterygian. Here, we present a detailed structural study with a histological and anatomical description of the vestigial lung of *L. chalumnae*, providing new insights into the arrested differentiation of this organ into a functional ABO.

## 2. Material and methods

Anatomical observations were made from new dissections of pre-dissected adult specimens CCC 3 (MNHN C3, isolated viscus from an adult male of 129 cm total length (TL), caught in Comoro Islands in 1953), CCC 24 (MNHN C22, isolated viscus from a female of 145 cm TL, caught in Comoro Islands in 1960), CCC 28 (MNHN C25, isolated viscus from a male of 130 cm TL, caught in Comoro Islands in 1961) and CCC 79 (MNHN C67, isolated viscus from a female of 163 cm TL, caught in Comoro Islands in 1972) deposited in the Collection of Comparative Anatomy of the Muséum national d'Histoire naturelle (MNHN). Further details of these specimens are provided elsewhere [24,25]. Since their capture, all of these specimens were stored in formalin solution (10%), except CCC 79, which is stored in alcohol after a short fixation in formalin.

CCC 3 and CCC 28 were also scanned by X-ray tomography at the Platform AST-RX of the MNHN, Paris (CAT scan), using a voltage of 245 kV and current of 430 mA for both specimens. A voxel size of 58.83 µm and 2200 views were acquired for CCC 3; and a voxel size of 54.24 µm and 2550 views were acquired for CCC 28.

CCC 202.1 (SAIAB 76199, early embryo of 4 cm TL found inside the female CCC 202, caught in Tanzania in 2005) and CCC 162.21 (ZSM 28409, late embryo of 35.6 cm TL found inside the female CCC 162, caught in Mozambique in 1991) were scanned using long-propagation phase-contrast synchrotron X-ray microtomography at the ID19 beamline of the European Synchrotron Radiation Facility (Grenoble, France). Voxel size is 6.5 µm for CCC 202.1 and 30.45 µm for CCC 162.21. Reconstructed volumes were reduced using binning of 2×2 pixels for CCC 202.1 and CCC 162.21 (13 µm and 60.90 µm, respectively). Both specimens were imaged with a high-quality pink beam using the ID19 W150 wiggler at a gap of 50 mm filtered by 2 mm of aluminium, 0.25 mm of copper, 0.2 mm of gold for CCC 202.1 and 0.25 of tungsten for CCC 162.21. The scintillator was a 250 mm-thick LuAG : Ce (lutetium–aluminium–garnet) crystal. The detector was a FreLoN 2 K charge-coupled device (CCD) camera mounted on a lens system. For a sufficient propagation phase-contrast effect, a distance of 3 m between the sample and the detector was used.

Images were reconstructed and exported into 16-bit TIFF stacks using the phoenix datos|x 2.0 reconstruction. Segmentation and three-dimensional rendering were made at the Palaeontology Imaging Unit of the MNHN Département Histoire de la Terre/UMR 7207 CR2P CNRS/MNHN/UPMC and at the Laboratório de Ictiologia Tempo e Espaço of the Universidade do Estado do Rio de Janeiro with the software MIMICS Innovation Suite 16.0 and 18.0 (Materialise).

Histological thin sections of the oesophagus and the vestigial lung (=oesophageal diverticulum) of *L. chalumnae* were prepared by Millot, Anthony and Robineau (1978) using azocarmine, haematoxylin/eosin and cajal colorations. These histological thin sections were prepared from specimen CCC 5 (MNHN C5, adult male of 127 cm TL, caught in Comoro Islands in 1954). This material is part of the historical material of extant coelacanths housed in the Collection of Comparative Anatomy of the MNHN, France, for which there is unfortunately no further detailed protocol archived.

Abbreviations: SAIAB, South African Institute for Aquatic Biodiversity, Grahamstown (South Africa); MNHN, Muséum national d'Histoire naturelle, Paris (France); ZSM, Zoologische Staatssammlung, München (Germany); CCC, Coelacanth Conservation Council.

## 3. Results

**Figure 1.**
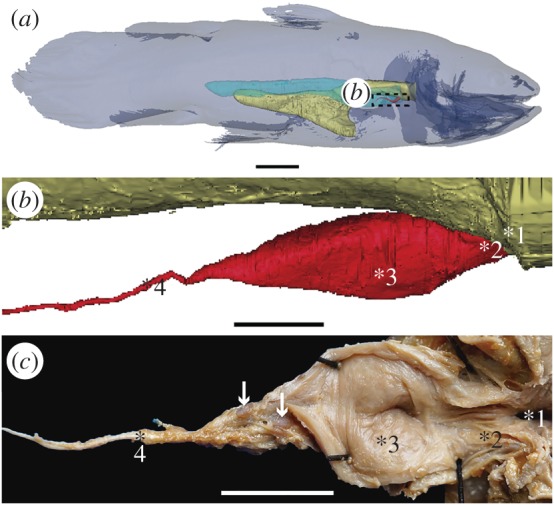
The vestigial lung of the extant coelacanth *Latimeria chalumnae*. (*a*) Three-dimensional reconstruction of the adult specimen CCC 22 (130 cm TL) in right lateral view. (*b*) Details of the three-dimensional reconstruction of the lung of the adult specimen CCC 28, corresponding to the boxed area in (*a*). (*c*) Partial dissection of the lung of the adult specimen CCC 3, exhibiting its lumen in the ventral view. Yellow, oesophagus and stomach; red, vestigial lung; blue, fatty organ. White arrows point to two hard but flexible plates. 1, 2, 3, 4 indicate the four successive areas of the vestigial lung. Scale bars, 10 cm (*a*); 1 cm (*b*); 0.5 cm (*c*).

**Figure 2.**
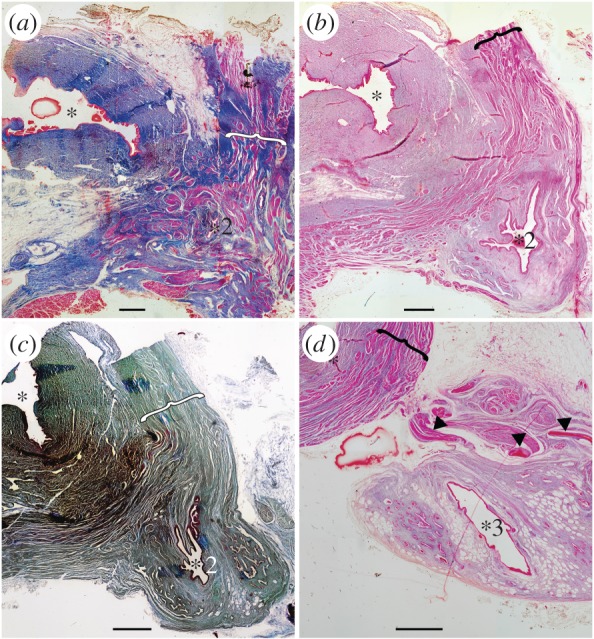
Histological thin sections of the anterior part of the vestigial lung, from adult specimen CCC 5. Oesophagus at the top left (asterisks indicate the lumen) and lung in the bottom right (numbered asterisks localize the lumen relative to the lung in figure 1*b,c*). (*a*) Vestigial lung at the level of its origin (asterisk 2 of figure 1) with disorganized muscle bundles (brackets) surrounding this organ. (*b*,*c*) Anterior portion (two successive sections) of the lung still in close proximity with the oesophagus, showing invaginations in the lung walls (asterisk 2 of figure 1). Muscle bundles distributed in an organized network (brackets). (*d*) Vestigial lung completely dissociated from the oesophagus (asterisk 3 of figure 1), presenting the clearly reduced invaginations of the lung walls. Arrowheads indicating the hard, but flexible, plates. Scales bar 0.1 cm (*a–d*). (*a*,*b*,*d*, azocarmin; *c*, cajal colorations).

**Figure 3.**
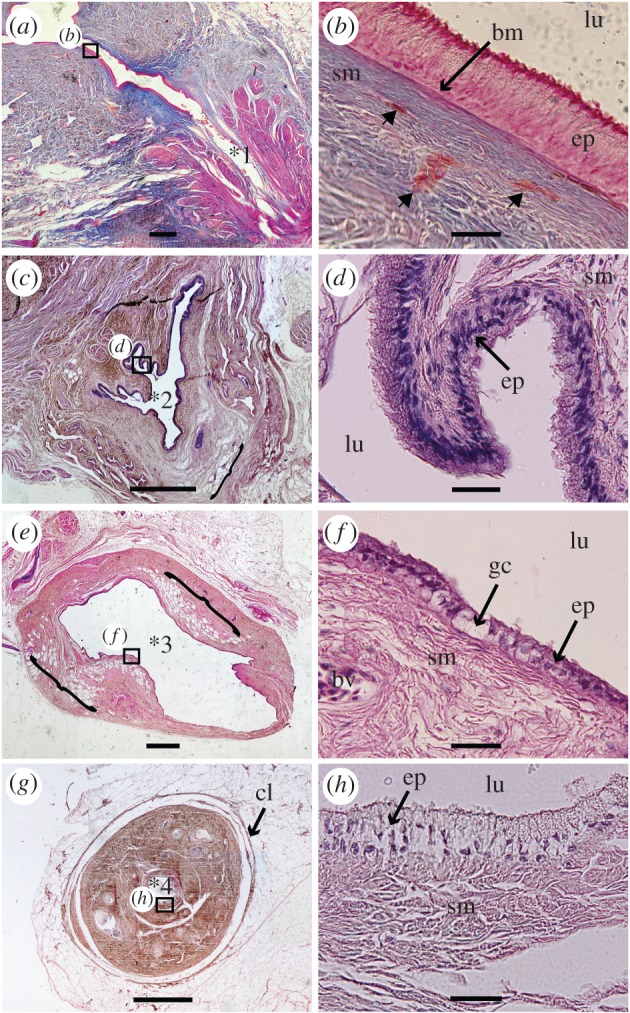
Histological thin sections of the vestigial lung of the adult specimen CCC 5. (*a*) The section shows the small and non-obliterated pneumatic duct. (*b*) Close-up of the oesophageal epithelium from the boxed area in (*a*), composed of a pseudostratified layer of epithelial ciliated cells. The epithelium (ep) lies on the submucosa constituted of intermingled muscular fibres, and blood vessels (arrowheads). (*c*) Section with the pleated epithelium of the lung, still linked to the oesophagus (figure 2*b,c*). (*d*) Close-up of the respiratory epithelium of the vestigial lung, from the boxed area in (*c*), showing the ciliated cells lying on the submucosa. (*e*) Section at the level of the middle portion of the vestigial lung fully dissociated from the oesophagus (figure 2*d*), still showing some minute invaginations and several areas with fat cells (brackets). (*f*) Close-up of the epithelium of the middle portion of the lung from the boxed area in (*e*) showing some rare ciliated cells and goblet cells. (*g*) Residual cord (asterisk 4 of figure 1). (*h*) Close-up of the epithelium of the residual cord from the boxed area in (*g*). Scale bars, 0.1 cm (*a*,*c*,*e*,*g*); 0.04 mm (*b*,*d*,*f*,*h*). Asterisks correspond to the same area indicated in figures 1 and 2. bm, basal membrane; bv, blood vessels; cl, conjunctive layers; ep, epithelium; gc, goblet cells; lu, lumen; sm, submucosa. (*a*,*b*, azocarmin; *c–h*, haematoxylin-eosin colorations).

**Figure 4.**
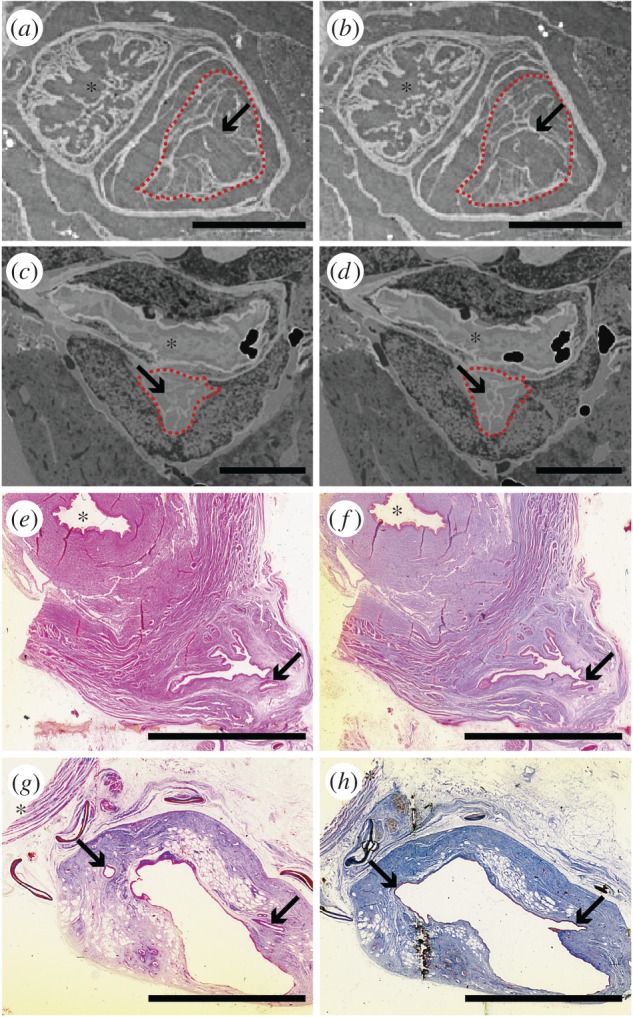
The compartmentalization of the extant coelacanth lung at different ontogenetic stages. (*a*,*b*) Sections of synchrotron X-ray microtomography of the early embryo CCC 202.1. (*c*,*d*) Sections of long-propagation phase-contrast synchrotron X-ray microtomography of the late embryo without yolk sac CCC 162.21. (*e–h*) Histological thin sections of the adult specimen CCC 5. Red dashed line, vestigial lung. Arrows indicating compartmentalized structures suggesting alveolation. Asterisks correspond to the oesophageal lumen. (*e*–*g*, azocarmin; *h*, cajal colorations). Scale bars, 1 mm (*a*,*b*); 5 mm (*c–h*).

Here, we describe the general morphology through partial dissections (figure 1), three-dimensional reconstructions (figure 1), histology (figures 2–4) and virtual thin-sections of long-propagation phase-contrast synchrotron X-ray microtomography (figure 4) of the lung of various developmental stages of the extant coelacanth *L. chalumnae*.

### 3.1. General morphology

The pulmonary complex of *L. chalumnae* is reduced to an oesophageal diverticulum originating in the ventromedial region of the anterior portion of the oesophagus. It is entirely included in the anteriormost part of a long tubular organ filled with fat (here called fatty organ) (figure 1*a*), also with a ventral origin in relation to the anterior part of the oesophagus [17,18]. Some major morphological features characterize the oesophageal diverticulum of *L. chalumnae* as a vestigial lung, such as its ventral position to the oesophagus and the presence of a non-obliterated opening between the oesophagus and the lung (figure 1*b,c*, asterisk 1) [18].

The vestigial lung of *L. chalumnae* is unpaired and present, in adult specimens, a unique morphology with a clear division in a short anterior chamber and a long and thin posterior residual cord (figure 1*b,c*, asterisks 3 and 4), highlighted by the presence of a septum observed from partial dissections and segmentation of high-resolution computerized axial tomography scans. Owing to the rarity of coelacanth specimens, particularly of their first ontogenetic stages, deposited in collections worldwide, we have not dissected embryo and juvenile individuals, and could not verify the presence of this septum in these ontogenetic stages.

The lung of adult individuals does not present alveolar septa, but only some invaginations in the anterior part of the anterior chamber (figures 1*c,* 2*a−d*, 3*c,e* and 4*e−h*), observed from dissections and histological thin sections described below. Virtual sections of high-quality tomography facilities of the earlier known embryo of *L. chalumnae* CCC 202.1 (4 cm TL), in which the lung exhibits some features still compatible with a potentially functional lung [18], and of the late embryo without yolk sac CCC 162.21 (35.6 cm TL) have evidenced the presence of compartmentalized structures throughout the length of the lung (figure 4*a−d*), suggesting the presence of alveolation in these first ontogenetic stages.

The vestigial lung has multiple conjunctive layers covering its whole length, a feature also observed from sections of high-resolution computerized axial tomography scans (from adult specimens CCC 3 and CCC 28). Inside the pulmonary sheath, there are dense layers of fat, more compact and thinner than those present in the fatty organ. The fatty organ presents also multiple sheaths covering its multiple fatty lobes. Glottal ridges have not been observed.

Based on thin sections, ‘pulmonary arteries’ have been previously described [23] for the pulmonary complex of *L. chalumnae*, but these structures have been recently identified [18] as small but dense plates that surround the vestigial lung of *Latimeria* (arrows and arrowheads in figures 1*c* and 2*d*, respectively). These small plates could be homologous to the calcified plates of Palaeozoic and Mesozoic coelacanth lungs [18]. During the development of this work, we have not identified the presence of true pulmonary arteries in *L. chalumnae*, probably due to the poor conservation of the pre-dissected material.

### 3.2. Histology

Regarding the histological and anatomical antero-posterior organization of the vestigial lung from adult specimens, that is from the region close to the oesophageal wall to the residual cord, we can define four areas (figures 1–3): the zone of the small pneumatic duct and the area immediately after it, still on the oesophageal wall (figure 3*a*, asterisk 1); the diverticulum area located just outside of the oesophagus (figure 2*a–c*; figure 3*c*, asterisk 2), but still linked to it (figures 2*a–c* and 3*c*); the anterior chamber where the vestigial lung is completely dissociated from the oesophagus (figures 2*d* and 3*e*, asterisk 3); and the residual cord (figure 3*g*, asterisk 4).

The vestigial lung at the level of its origin exhibits a small and pleated lumen, sharing the same features of the *oesophagus mucosa* and *submucosa* (figure 2*a*). The *muscularis mucosa* presents transversal and longitudinal muscle bundles in a diverse organization, depending on the degree of development. In this section, there are disorganized muscle bundles surrounding the lung (brackets in figure 2*a*). All sections of the oesophagus present a mucosa composed of a pseudostratified epithelium with tall columnar ciliated cells intercalated by goblet cells and a submucosa with collagenous and elastic fibres (figure 3*a,b*). The presence of a small, unobstructed and muscular duct connecting the oesophagus with the vestigial lung is highlighted by histological thin sections and dissections (figures 1*c* and 3*a*). The muscles that adjoin the glottis (figure 3*a*) may have had the function of control of the glottis opening during air swallow and expiration, also described and suggested for the ABOs of *Lepisosteus oculatus* and for the lung of *Protopterus dolloi* [3,26]. This function has disappeared in the extant coelacanth *L. chalumnae* that inhabits moderately deep waters.

The second area is represented by the anterior portion of the lung still in close proximity with the oesophagus. It reveals the presence of a pseudostratified and pleated epithelium, composed of tall columnar ciliated cells intercalated by goblet cells (figures 2*b*,*c*, 3*c*,*d* and 4*e,f*). The submucosa of the lung presents collagenous and elastic fibres, numerous blood vessels and a small number of vacuolar sectors for fat reserve (brackets in figure 3*c*). The *muscularis mucosa* presents muscle bundles distributed in an organized network, beginning the differentiation between the oesophagus and the lung (brackets in figure 2*b,c*).

In the third area, the vestigial lung is completely dissociated from the oesophagus (figures 2*d* and 3*e*). The lumen of the anterior chamber of the vestigial lung is almost not pleated (figure 4*g,h*) and the mucosa presents a reduced number of tall columnar, and sometimes cuboidal, ciliated cells in the mucosa intercalating with goblet cells (figures 2*d* and 3*e*). The submucosa of this portion is composed of collagenous and elastic fibres, numerous blood vessels and a greater amount of vacuolar sectors for fat reserve, indicating the beginning of the vestigial feature (figure 3*e,f*). The diameter of the diverticulum lumen in this area increases over the previous histological thin section.

The most posterior region corresponds to the residual cord [23] and presents a fibrous submucosa with some blood vessels, a mucosa with goblet cells and with a reduced number of ciliated cells, as well as an extremely reduced lumen (figure 3*g,h*).

## 4. Discussion

Among the extant fauna, many aquatic vertebrate taxa—such as aquatic tetrapods, sarcopterygian fishes[18–21,26–30], polypterids [14,16,31,32], holosteans [2,3,33–35] and some teleosts [36,37]—are able to make gas exchange employing many different ABOs [1,3].

Although morphologically distinguishable, lungs and physostomous gas bladders of aquatic vertebrates display some structures with analogous function that allow, or increase, the gas exchange, such as: anteriorly non-obliterated duct; respiratory epithelium (composed of ciliated cells, goblet cells and pneumocytes); compartmentalization (including the presence of alveoli for some taxa, such as the marine tetrapods and lungfishes *Neoceratodus*, *Protopterus* and *Lepidosiren*) [1,19,38]; and rich vascularization.

The extant coelacanth lung is unpaired, well vascularized and originated from the ventral portion of the oesophagus by the presence of a non-obliterated opening [18]. Although this lung exhibits some respiratory features by the presence of ciliated cells intercalated by goblet cells in the adult specimens here analysed, it lacks compartmentalization and pneumocytes.

The histological arrangement of the pleated anteriormost portion of the anterior chamber of the lung of adult specimens is similar to the histology of the oesophagus, by the presence of a pleated epithelium composed of ciliated cells intercalated by goblet cells (figures 1*c*, 2*b*,*c* and 3*c*,*d*), and, therefore, reveals it as a structure derived from the oesophagus with an arrested differentiation into an ABO. The presence of invaginations in the anterior chamber of the vestigial lung of *Latimeria* in this adult stage is the only characteristic that can be interpreted as a vestige of alveolization (figure 4). By contrast, embryos CCC 202.1 and CCC 162.21 present compartmentalized structures throughout the length of the lung (figure 4*a–d*). This structural organization may suggest the presence of alveolation in these ontogenetic stages and confirms the marked reduction of the lung throughout different developmental stages.

Histological features of the vestigial lung of the adult *L. chalumnae* are structurally similar to the conducting portion (e.g. central duct) of the respiratory systems of some other functional ABOs of actinopterygians and sarcopterygians, for instance in the holostean *Lepisosteus* and the lungfish *Protopterus* [3,26,31], and stores functional features for conduction of the air. Nonetheless, extant air-breathing actinopterygian and sarcopterygian fishes that have functional ABOs present, in general, the respiratory portion constituted by compartments and pneumocytes [3,26].

The vestigial stage of the lung of *L. chalumnae* has been recently interpreted by the negative allometric growth of this organ when compared with the growth of the fatty organ and the TL [18]. The vestigial feature of this organ is here supported also by anatomical and histological features observed in adult specimens, such as the absence of compartments and pneumocytes. Although functional single-chambered lungs have been reported for polypterids [1,31], amphibians and the majority of adult lepidosaurs, the simplification of this organ seems to have appeared secondarily, and all the remaining amniotes retain multichambered lungs, which increases the functional surface and hence offers advantages for efficient respiration in terrestrial environments [39]. Some fossil coelacanth genera (e.g. *Axelrodichthys* from the Cretaceous) show a well-developed calcified lung with one or two constrictions [17], suggesting a multichambered lung.

The negative allometric growth added to the histological and anatomical features of the lung, with an arrested differentiation for a functional respiratory complex, offers new insights into the vestigial stage of this organ in the extant coelacanth *L. chalumnae*, which inhabits moderately deep waters, and whose main oxygen supply is provided through gills [40].

## 5. Conclusion

The extant coelacanth lung presents a vestigial feature at the adult stage, morphologically represented by a reduced size, a short anterior chamber and a proportionally long residual cord. Our detailed structural study and the previously described allometric growth of this lung support the arrested differentiation of this organ to a functional respiratory complex. This vestigial lung is structurally similar to the conducting portion of other ABOs, but lacks true compartments and pneumocytes, the main components of the respiratory portion in osteichthyans.

## Supplementary Material

Interactive 3D PDF model of *Latimeria chalumnae* oesophagus and vestigial lung. Three-dimensional reconstruction of the lung and oesophagus of the adult specimen CCC 28. Yellow, oesophagus; red, vestigial lung.

## Data accessibility

The datasets supporting this article have been uploaded as part of the electronic supplementary material.
