# Supplementary material for: Lung anatomy and histology of the extant coelacanth shed light on the loss of air-breathing during deep-water adaptation in actinistians
Source: R Soc Open Sci. 2017 Mar 8;4(3):161030. doi: 10.1098/rsos.161030 (PMC5383850; doi:10.1098/rsos.161030)
Supplement: Interactive 3D PDF model of Latimeria chalumnae oesophagus and vestigial lung. Three-dimensional reconstruction of the lung and oesophagus of the adult specimen CCC 28. Yellow, oesophagus; red, vestigial lung. [file rsos161030supp1.pdf]

ROYAL SOCIETY OPEN SCIENCE
Article title: Lung anatomy and histology of the extant coelacanth shed light on the loss of air-breathing during deep-water adaptation in actinistians
Authors: Camila Cupello, François J. Meunier, Marc Herbin, Gaël Clément, Paulo M. Brito

Latimeria chalumnae CCC 28
